# Items

**Published:** 1905-01

**Authors:** 


### ITEMS.

MESSRS. BATTLE & COMPANY, Saint Louis, announce that they recently issued the fourth number in a series of twelve illustrations of intestinal parasites, which they will send free to physicians on application.

---

THE Pope Bicycle Daily Calendar for 1905 contains a memorandum leaf for every day in the year, and 365 original sayings in favor of good roads, good health, outdoor exercise, and that great vehicle of health-giving, the modern bicycle, by our most eminent living men of marked accomplishment. The calendar is free at Pope Manufacturing Company's stores or any of our readers can obtain it by sending five 2-cent stamps to Pope Manufacturing Company, Hartford, Conn., or 143 Sigel street, Chicago, Ill.

---

MESSRS. M. J. BREITENBACH COMPANY'S Physicians' Daily Memorandum, the most convenient and elaborate desk reminder that has been prepared for medical men, is issued for the year 1905. It will be furnished on application and eight cents postage.

---

MR. CHARLES MARCHAND, president of the Drevet Manufacturing Company, has invented and patented a safety valve stopper which is intended to reduce bottle breakage very materially. He proposes to use flint glass containers for hydrogen peroxide and cork them with Marchand's safety valve stopper. This will reduce the danger of breakage to a minimum—as near the preventable stage as can be attained. This is a commendable improvement and will materially increase the sales of Marchand's hydrogen peroxide.

---

MELLIN'S FOOD COMPANY has received the Grand Prize for Mellin's Food, which is the highest award of the World's Fair at Saint Louis.

